# Eruptive Seborrheic Keratoses Are Associated With a Co-Occurring Malignancy in the Majority of Reported Cases: A Systematic Review

**DOI:** 10.1177/12034754211035124

**Published:** 2021-11-28

**Authors:** Sara Mirali, Asfandyar Mufti, Rafael Paolo Lansang, Muskaan Sachdeva, Jensen Yeung

**Affiliations:** 1 12366 Faculty of Medicine, University of Toronto, Toronto, ON, Canada; 2 7938 Department of Dermatology, University of Toronto, Toronto, ON, Canada; 3 62703 Faculty of Health Sciences, McMaster University, Hamilton, ON, Canada; 4 Sunnybrook Health Sciences Centre, Toronto, Ontario, Canada; 5 Women’s College Hospital, Toronto, ON, Canada; 6 Probity Medical Research Inc., Waterloo, ON, Canada

**Keywords:** Eruptive seborrheic keratoses, ESK, lichenoid keratoses, Leser-Trelat, cancer, adenocarcinoma, systematic review

## Abstract

**Background:**

Eruptive seborrheic keratoses (ESK) is a benign skin condition that has been associated with malignant and nonmalignant diseases. We conducted a systematic review of reported cases of ESK to identify and summarize associated comorbidities.

**Methods:**

MEDLINE and Embase were searched from database inception (1946) to July 31, 2020 for original articles describing ESK with or without a co-occurring condition. Subject demographics, as well as details of ESK and associated diagnoses were extracted from 76 articles (70 case reports, 3 case series, 3 case control studies) representing 92 patients.

**Results:**

In total, 76.1% (*n* = 70/92) of patients with ESK had a co-occurring malignancy, 4.3% (*n* = 4/92) presented with a nonmalignant condition, 9.8% (*n* = 9/92) experienced ESK as an adverse drug reaction, and 9.8% (*n* = 9/92) did not report any underlying medical condition. ESK preceded a cancer diagnosis in 76.1% (*n* = 70/92) of patients with a mean latency period of 4.0 months (range: 0.25-9 months). The most common malignancies associated with ESK were cutaneous T-cell lymphoma (*n* = 10/70, 14.3%) and gastrointestinal adenocarcinoma (*n* = 9/70, 12.9%). ESK preceded nonmalignant conditions or no disease in 14.1% (*n* = 13/92) of patients with a mean latency period of 3.1 months (range: 0.75-6 months). Drug-induced ESK occurred in 9.8% (*n* = 9/92) of patients with a mean latency period of 7.1 weeks after changing medication.

**Conclusion:**

Although the role of ESK as a paraneoplastic cutaneous marker is debated, healthcare providers should consider screening for underlying malignancy in patients presenting with ESK. Larger studies are needed to confirm its role as a marker for disease.

## Introduction

Eruptive seborrheic keratoses (ESK) is a rare dermatological disorder characterized by the rapid appearance of several benign pigmented skin lesions. ESK presents as well-demarcated round or oval lesions with a verrucous surface. It is diagnosed clinically although in rare cases, a biopsy can be used to differentiate ESK from other benign and malignant lesions.^1,2^ Histopathologic examination shows proliferation of keratinocytes with keratin-filled cysts.^3^ Treatment is not required but can be considered if the lesions are symptomatic or for cosmetic reasons. Treatment options include cryotherapy, shave excision, electrodessication, hydrogen peroxide, and laser treatment.^3-6^

Although ESK is a benign condition, it has been associated with malignant and nonmalignant conditions, including gastrointestinal adenocarcinoma, viral infection, and inflammatory skin conditions.^7-9^ ESK has also been reported as an adverse drug reaction.^10^ Although there have been clinical reviews about ESK, to our knowledge there have been no systematic reviews to-date.^8,11^

Given its potential role as an early marker of cancer or other illnesses, it is important for healthcare workers to promptly identify ESK and to understand its associated conditions. The goal of this systematic review is to identify and summarize malignant and nonmalignant conditions in patients with ESK. The secondary objective is to report outcomes and characteristics of ESK after medication use.

## Methods

### Search Strategy

MEDLINE and Embase were searched from database inception (1946) to July 31, 2020 using the OVID interface in accordance with the Preferred Reporting Items for Systematic Review and Meta-Analysis guidelines.^12^ No language restrictions and no publication period restrictions were applied. Variations of the following keywords were used for the search: “Leser-Trelat”, “Stucco keratoses,” “Seborrheic keratoses,” or “Lichenoid keratosis” in combination with “eruptive,” “abrupt,” explosive,” and “sudden.”

### Study Eligibility Criteria

Original articles that described ESK in association with other medical conditions were included if they met the following criteria: (1) included patients diagnosed with ESK, (2) if latency period was described, only patients with an ESK latency period of less than a year were included, and (3) were observational (ie, case reports, case series, cross-sectional, or cohort studies). The following studies were excluded: (1) animal and in vitro studies, (2) systematic reviews or review articles, and (3) commentaries.

### Study Selection

The literature search was conducted by 2 independent reviewers (S.M. and R.P.L.) who reviewed the list of titles, abstracts, and full texts to identify eligible studies. Discrepancies between the 2 reviewers were discussed and if a consensus was not reached, a 3rd reviewer (A.M.) was consulted and a final decision was made. Reference lists from included articles were reviewed to identify additional studies that were not found in the initial search.

### Data Collection and Analysis

Data collection was independently conducted by 2 reviewers (S.M. and R.P.L.), who reviewed and extracted data from included studies. Discrepancies between the 2 reviewers were discussed and if a consensus was not reached, a 3rd reviewer (A.M.) was consulted and a final decision was made. Outcome measures are defined in Supplemental File 1. This review used a descriptive analysis of observational studies.

### Quality Assessment

The level of evidence for all include articles were assessed by S.M. and R.P.L. according to the Oxford Centre for Evidence-Based Medicine.^13^ Quality appraisal was performed by S.M. and R.P.L. using Murad et al. (case reports and case series) and the Newcastle-Ottawa scale (case control studies).^14,15^ If a consensus was not reached, a 3rd reviewer (A.M.) was consulted and a final decision was made. (Supplemental Tables S1 and S2).

In total, 26 case reports and case series were scored as good (5 + points), 32 fair (3, 4 points), and 15 poor (1, 2 points) using the Murad et al. scale. All included case control studies scored as good (6 + points) using the Newcastle-Ottawa scale.^14,15^

## Results

### Study Demographics and Patient Characteristics

After screening the titles and abstracts of 366 articles, the full texts of 253 studies were reviewed. In total, 76 studies met the inclusion criteria and were used for data collection and analysis (Supplementary Figure S1, Table 1, Supplemental Tables S3-S5). Overall, 92.1% (*n* = 70/76) of studies had a level of evidence of 5, 6.6% (*n* = 5/76) had a level of evidence of 4, and 1.3% (*n* = 1/76) had a level of evidence of 3b. Of the 92 patients that were included in the analysis, the mean age was 60.3 years (range: 13-92 years) and among patients in whom sex was reported, 47.3% (*n* = 35/74) were female and 52.7% (*n* = 39/74) were male. In total, 76.1% (*n* = 70/92) of patients with ESK had a co-occurring malignancy, 4.3% (*n* = 4/92) had a coinciding nonmalignant condition, 9.8% (*n* = 9/92) did not report any underlying medical condition, and 9.8% (*n* = 9/92) experienced ESK as an adverse drug reaction.

**Table 1:** Summary of Included Cases of Drug-Induced ESK.

Study information		Demographic information			Drug information				ESK resolution			
Study design, level of evidence	Sample size	Age, sex	Associated condition(s)	Comorbidities	Treatment for associated condition(s)	Patient self-report?	Latency (weeks after starting treatment)	Associated medication stopped	Correlation between stopping medication and ESK improvement	ESK resolution period (months)	Recovery of ESK	Naranjo ADR Scale
CR,^1^ 5	1	60, NR	Immunosuppression	Heart transplant	Prednisone 5 mg daily, ciclosporin 60 mg daily	Y	3	NR	NR	NR	NR	3
CR,^2^ 5	1	71, *M*	Rheumatoid arthritis	NR	Adalimumab	Y	10	Yes	Yes	12	Complete	4
CR,^3^ 5	1	22, *M*	Intracranial tumor	NR	Dexamethasone, radiotherapy	N	9	Yes	No	NR	Complete	1
CS,^4^ 4	2	56, *F*	Metastatic breast adenocarcinoma	NR	Diethylstilbestrol, surgery	Unclear	4	Yes	No	NR	None	3
		58, *F*	Breast adenocarcinoma	NR	Conjugated estrogens, surgery		8	Yes	No	NR	None	3
CR,^5^ 5	1	65, *F*	Hepatitis C	Depression, basal cell carcinoma	Interferon, ribavirin, telaprevir	Y	8	Telaprevir was stopped	Yes	NR	Complete	4
CR,^6^ 5	1	40, *F*	Metastatic neurofibrosarcoma	NR	Mitomycin (8 mg/m^2^), adriamycin (40 mg/m2), cisplatin (60 mg/m2)	Y	3	Yes	Yes	NR	Partial	4
CR,^7^ 5	1	73, *M*	Metastatic colorectal adenocarcinoma	NR	Panitumumab and folinic acid, fluorouracil, oxaliplatin	Unclear	16	Yes	Yes	1	Complete	2
CR,^8^ 5	1	78, *M*	NR	NR	Generic gluconate, ranitidine (switched from brand name)	Y	3	Yes	No	12	Complete	3

Abbreviations: ADR, adverse drug reaction; CR, case report; CS, case series; ESK, eruptive seborrheic keratoses; F, female; M, male; NR, not reported.

**References**

### Malignant Conditions

Of the 83 patients who reported ESK with an associated condition, the majority (84.3%, *n* = 70/83) had an underlying neoplastic condition with a mean age of 62.8 (range: 20-92) years (Supplemental Table S3). Patients were diagnosed with solid malignancies (*n* = 48/70, 68.6%), hematological malignancies (*n* = 12/70, 17.1%), both (*n* = 2/70, 2.9%), or specifics were not reported (*n* = 8/70, 11.4%). The most common hematological malignancy was cutaneous T-cell lymphoma (*n* = 10/70, 14.3%) and the most common solid tumor was gastrointestinal adenocarcinoma (*n* = 9/70, 12.9%). On average, ESK appeared 4.0 months (range: 0.25-9 months) before a cancer diagnosis. Comorbidities were reported in 32.9% (*n* = 23/70) of cancer patients with the most common being hypertension = 5/23, 21.7%), obesity (*n* = 5/23, 21.7%), and diabetes mellitus (*n* = 4/23, 17.4%). Following cancer treatment, 19 patients experienced improvement in their ESK lesions. Among these patients, complete resolution was reported in 42.1% (*n* = 8/19) of patients with a mean resolution period of 6.7 months and partial resolution was reported in 57.9% (*n* = 11/19) of patients with a mean resolution period of 3.4 months.

Despite the temporal relationship observed between the development of ESK and a cancer diagnosis in smaller studies, larger case control studies found no relationship between ESK and the incidence of cancer (Supplemental Table S5). ESK was a rare occurrence amongst both cancer patients and controls with a prevalence rate of 0.3% to 2.3% and 0.0% to 8.0%, respectively. Given the low incidence rate, an association between ESK and malignancy could not be made.^16-18^

### Nonmalignant Conditions

ESK coincided with nonmalignant diseases or no disease in 14.1% (*n* = 13/92) of patients with a mean age of 44.9 years (range: 13-74 years). The mean latency period between ESK onset and presentation was 3.1 months (range: 0.75-6 months). These patients presented with various diseases. Specifically, ESK co-occurred with erythrodermic pityriasis rubra pilaris (*n* = 1), diabetes mellitus (*n* = 1), secondary syphilis (*n* =1) and human immunodeficiency virus (*n* = 1).

### ESK Eruptions After Medications Use

ESK was described as an adverse effect to medication in 9.8% (*n* = 9/92) of patients (Table 1). Of these patients, the mean age was 58.0 years (range: 22-78) and the mean latency period was 7.1 weeks after changing medication. The mean Naranjo score was 3, indicating possible association. ESK was noted after treatment with corticosteroids in 22.2% (*n* = 2/9) of patients and after starting synthetic estrogens in 22.2% (*n* = 2/9). A prior cancer diagnosis was made in 55.6% (*n* = 5/9) of patients.

## Discussion

ESK is a rare, benign, and underdiagnosed skin disease that may be associated with malignant or nonmalignant conditions.^16^ Our data found that 76.1% (*n* = 70/92) of reported cases occurred in patients that were subsequently diagnosed with a malignancy. In accordance with previous literature, we found that ESK was most commonly associated with cutaneous T-cell lymphoma and gastric adenocarcinoma.^8^ To support their uncontrolled proliferation, cancer cells produce growth factors which bind to receptors on cell membranes that drive intracellular growth signalling. Activation of the epidermal growth factor receptor (EGFR) family is a hallmark of many cancers.^19^ EGFR are found in keratinocytes and can be activated by epidermal growth factor (EGF) or transforming growth factor α (TGF-α).^20,21^ Growth factor secretion from the tumor may drive keratinocyte growth via EGFR signaling, resulting in ESK. Supporting this hypothesis, seborrheic keratoses lesions from a melanoma patient showed increased EGFR levels compared to control skin.^21^ Although increased EGF levels were not observed in ESK patients, TGF-α levels were elevated and became undetectable after treatment, suggesting that TGF-α is the main driver of ESK.^21,22^ In addition, TGF-α have previously been shown to be upregulated in precursor lesions of gastric carcinomas.^23^ Thus, it is plausible that ESK may be correlated with EGFR mediated proliferation of keratinocytes caused by growth factors secreted by the tumor.

ESK’s role as a paraneoplastic cutaneous marker is debated in the literature. Although case control studies found no association between ESK and malignancy, the incidence of ESK was low in both patient and control groups. Given the small sample size, no conclusions could be made on the association between ESK and malignancy.^16-18^ Another criticism of ESK’s link to malignancy is that the incidence of both seborrheic keratoses and cancer is higher in older patients.^24^ While the majority of patients were elderly in our analysis, we found that 31.4% (*n* = 22/70) of patients who had ESK prior to a cancer diagnosis were under the age of 60. Moreover, 27.1% (*n* = 19/70) of cancer patients reported resolution of ESK following treatment, although this did not always correlate with regression of the tumor. Of the 4 reports that noted recurrence of ESK in cancer patients, all were associated with a relapse of the primary cancer or a new malignancy.^25-28^ These data suggest that there is a temporal relationship between ESK and malignancy that may not be strictly due to increased age.

^29-31^ ^30,31^

Our systematic review has several limitations. Firstly, the definition of ESK varied between studies and most articles were case reports or case series, with an overall mean evidence level of 4.9.^13^ In some cases, a diagnosis of ESK was made by visual appearance alone and images were not available for our assessment. In 58.7% (*n* = 54/92) of cases, the ESK latency were based on patient self-reports, which may be unreliable. Moreover, the number of ESK patients with no associated disease may be underestimated because of reporting bias. Additionally, other reports have noted inflammation of preexisting seborrheic keratoses following treatment with chemotherapy.^32-35^ Although we excluded these reports from our analysis on drug-induced ESK, it is possible that patients noticed an inflammation of previously unrecognized seborrheic keratoses, rather than the eruption of new ones. Of the included studies describing drug induced ESK, none prove causality (mean Naranjo score: 3, indicating possible association). It is difficult to attribute a link between ESK and treatment as 55.6% (*n* = 5/9) of patients who developed drug induced ESK were also diagnosed with cancer. Moreover, this may also have contributed to misclassification bias. Finally, data on ESK resolution was unavailable in 68.5% (*n* = 63/92) of patients, which limits our analysis.

Despite these limitations, our study demonstrates that ESK precedes malignant and nonmalignant conditions and has also been reported as an adverse drug reaction. Results from future studies will be important in determining if there is a causative link between ESK and disease. Further studies are required to understand the prevalence of ESK and its associated conditions.

## Supplemental Material

Online supplementary file 1 - Supplemental material for Eruptive Seborrheic Keratoses Are Associated With a Co-Occurring Malignancy in the Majority of Reported Cases: A Systematic Review Click here for additional data file. Supplemental material, Online supplementary file 1, for Eruptive Seborrheic Keratoses Are Associated With a Co-Occurring Malignancy in the Majority of Reported Cases: A Systematic Review by Sara Mirali, Asfandyar Mufti, Rafael Paolo Lansang, Muskaan Sachdeva and Jensen Yeung in Journal of Cutaneous Medicine and Surgery

## References

[1] Izikson L. Sober AJ. Mihm MC. Zembowicz A . Prevalence of melanoma clinically resembling seborrheic keratosis: analysis of 9204 cases. Arch Dermatol. 2002;138(12):1562-1566. 10.1001/archderm.138.12.1562 12472343

[2] Longo C. Moscarella E. Piana S et al. Not all lesions with a verrucous surface are seborrheic keratoses. J Am Acad Dermatol. 2014;70(6):e121-123. 10.1016/j.jaad.2013.10.042 24831328

[3] Hafner C. Vogt T . Seborrheic keratosis. J Dtsch Dermatol Ges. 2008;6(8):664-677. 10.1111/j.1610-0387.2008.06788.x 18801147

[bibr4-12034754211035124] Wood LD. Stucki JK. Hollenbeak CS. Miller JJ . Effectiveness of cryosurgery vs curettage in the treatment of seborrheic keratoses. JAMA Dermatol. 2013;149(1):108-109. 10.1001/2013.jamadermatol.275 23324775

[bibr5-12034754211035124] Ethington E. Mitri A. Surprenant D et al. Patient preferences and comparative outcomes regarding cryosurgery versus Electrodesiccation in the removal of truncal seborrheic keratoses. J Clin Aesthet Dermatol. 2019;12(9):E53-E56. 31641420 PMC6777698

[6] Kim YK. Kim D-Y. Lee SJ. Chung WS. Cho SB . Therapeutic efficacy of long-pulsed 755-nm alexandrite laser for seborrheic keratoses. J Eur Acad Dermatol Venereol. 2014;28(8):1007-1011. 10.1111/jdv.12231 23909912

[7] Lynam K. Fahs F. Daveluy S . Sudden onset psoriasis causing eruptive seborrheic keratoses. Int J Dermatol. 2020;59(7):e257-e259. 10.1111/ijd.14822 32060898

[bibr8-12034754211035124] Schwartz RA . Sign of Leser-Trelat. [Review] [129 refs]. 1996;1(1):88-95. 10.1016/S0190-9622(96)90502-2 8682971

[9] Inamadar AC. Palit A . Eruptive seborrhoeic keratosis in human immunodeficiency virus infection: a coincidence or the sign of leser-trélat? Br J Dermatol. 2003;149(2):435-436. 10.1046/j.1365-2133.2003.05463.x 12932267

[10] Williams JV. Helm KF. Long D . Chemotherapy-induced inflammation in seborrheic keratoses mimicking disseminated herpes zoster. J Am Acad Dermatol. 1999;40(4):643-644. 10.1016/s0190-9622(99)70455-x 10188692

[11] Yamamoto T . Leser-trelat sign: current observations. Expert Rev Dermatol. 2013;8(5):541-546. 10.1586/17469872.2013.835926

[12] Moher D Liberati A Tetzlaff J Altman DG Group P PRISMA Group . Preferred reporting items for systematic reviews and meta-analyses: the PRISMA statement. BMJ. 2009;339:b2535. 10.1136/bmj.b2535 19622551 PMC2714657

[13] Oxford Centre for Evidence-based Medicine – Levels of Evidence. 2020 . https://www.cebm.net/2009/06/oxford-centre-evidence-based-medicine-levels-evidence-march-2009

[14] Murad MH. Sultan S. Haffar S. Bazerbachi F . Methodological quality and synthesis of case series and case reports. BMJ Evid Based Med. 2018;23(2):60-63. 10.1136/bmjebm-2017-110853 29420178 PMC6234235

[15] Wells G. Shea B. O’Connell D et al. The Newcastle-Ottawa Scale (NOS) for assessing the quality of nonrandomised studies in meta-analyses. http://www.ohri.ca/programs/clinical_epidemiology/oxford.asp

[16] Lindelöf B. Sigurgeirsson B. Melander S . Seborrheic keratoses and cancer. J Am Acad Dermatol. 1992;26(6):947-950. 10.1016/0190-9622(92)70139-7 1535080

[bibr17-12034754211035124] Fink AM. Filz D. Krajnik G. Jurecka W. Ludwig H. Steiner A . Seborrhoeic keratoses in patients with internal malignancies: a case-control study with prospective accrual of patients. J Eur Acad Dermatol Venereol. 2009;23(11):1316-1319. 10.1111/j.1468-3083.2009.03163.x 19309432

[18] Insuasty J. Diaz L. Berbeo M. Ballesteros Z. Mantilla A . RM-043 eruptive seborrheic keratosis (ESK) and its association with gastrointestinal cancer (GIC): a case-control study and meta-analysis. Annals of Oncology. 2016;27(Supplement 2):ii95-ii96. 10.1093/annonc/mdw201.40

[19] Ciardiello F. Tortora G . EGFRantagonists in cancer treatment. N Engl J Med. 2008;358(11):1160-1174. 10.1056/NEJMra0707704 18337605

[20] Nanney LB. Magid M. Stoscheck CM. King LE . Comparison of epidermal growth factor binding and receptor distribution in normal human epidermis and epidermal appendages. J Invest Dermatol. 1984;83(5):385-393. 10.1111/1523-1747.ep12264708 6092481

[21] Ellis DL. Kafka SP. Chow JC et al. Melanoma, growth factors, acanthosis nigricans, the sign of leser-trelat, and multiple acrochordons. A possible role for alpha-transforming growth factor in cutaneous paraneoplastic syndromes. N Engl J Med. 1987;317(25):1582-1587. 10.1056/NEJM198712173172506 2825016

[22] Curry SS. King LE . The sign of Leser-Trelat. Report of a case with adenocarcinoma of the duodenum. Arch Dermatol. 1980;116(9):1059-1060. 10.1001/archderm.116.9.1059 6968179

[23] Filipe MI. Osborn M. Linehan J. Sanidas E. Brito MJ. Jankowski J . Expression of transforming growth factor alpha, epidermal growth factor receptor and epidermal growth factor in precursor lesions to gastric carcinoma. Br J Cancer. 1995;71(1):30-36. 10.1038/bjc.1995.7 7819044 PMC2033456

[24] Brauer J. Happle R. Gieler U. Effendy I . The sign of leser-trelat: fact or myth? J Eur Acad Dermatol Venerol. 1992;1(1):77-80. 10.1111/j.1468-3083.1992.tb00703.x

[25] Ohashi N. Hidaka N . Pancreatic carcinoma associated with the leser-trelat sign. Int J Pancreatol. 1997;22(2):155-160. 10.1007/BF02787475 9387039

[bibr26-12034754211035124] Fetil E. Ozkan S. Gürler N. Kuşku E. Arda F. Güneş AT . Recurrent leser-trelat sign associated with two malignancies. Dermatology. 2002;204(3):254-255. 10.1159/000057894 12037460

[bibr27-12034754211035124] Ikari Y. Ohkura M. Morita M. Seki K. Kubota Y. Mizoguchi M . Leser-trelat sign associated with Sezary syndrome. J Dermatol. 1995;22(1):62-67. 10.1111/j.1346-8138.1995.tb03343.x 7897028

[28] Liddell K. White JE. Caldwell IW . Seborrhoeic keratoses and carcinoma of the large bowel. Three cases exhibiting the sign of Lester-trélat. Br J Dermatol. 1975;92(4):449-452. 10.1111/j.1365-2133.1975.tb03107.x 125606

[29] Al Ghazal P. Körber A. Klode J. Dissemond J . Leser-trelat sign and breast cancer. Lancet. 2013;381(9878):1653. 10.1016/S0140-6736(12)61257-4 23201213

[bibr30-12034754211035124] Flugman SL. McClain SA. Clark RA . Transient eruptive seborrheic keratoses associated with erythrodermic psoriasis and erythrodermic drug eruption: report of two cases. J Am Acad Dermatol. 2001;45(6 Suppl):S212-S214. 10.1067/mjd.2001.103641 11712062

[31] Chadwick PW. Heymann WR . Eruptive seborrheic keratoses secondary to telaprevir-related dermatitis. Cutis. 2016;98(2):E20-21. 27622264

[32] Kechijian P. Sadick NS. Mariglio J. Schulman P . Cytarabine-induced inflammation in the seborrheic keratoses of leser-trelat. Ann Intern Med. 1979;91(6):868-869. 10.7326/0003-4819-91-6-868 293142

[bibr33-12034754211035124] Patton T. Zirwas M. Nieland-Fisher N. Jukic D . Inflammation of seborrheic keratoses caused by cytarabine: a pseudo sign of leser-trelat. J Drugs Dermatol. 2004;3(5):565-566. 15552612

[bibr34-12034754211035124] Aamir S. Ullah Z. Iqbal Z. Khan AA. Yaqub F. Malik K . Cutaneous manifestations of interferon alfa and ribavirin for hepatitis C. J Pak Assoc Dermatol. 2008;18:14-20.

[35] Kowalzick L. Truhm B. Theilig M. Eickenscheidt L. Neuser H . Pseudo-sign of leser-trelat: acute inflammation of seborrheic keratoses during chemotherapy with docetaxel. [German]. 2013;10:397-399.

