# Supplementary material for: Eruptive Seborrheic Keratoses Are Associated With a Co-Occurring Malignancy in the Majority of Reported Cases: A Systematic Review
Source: J Cutan Med Surg. 2021 Nov 28;26(1):57–62. doi: 10.1177/12034754211035124 (PMC8750131; doi:10.1177/12034754211035124)
Supplement: Online supplementary file 1 - Supplemental material for Eruptive Seborrheic Keratoses Are Associated With a Co-Occurring Malignancy in the Majority of Reported Cases: A Systematic Review [file sj-pdf-1-cms-10.1177_12034754211035124.pdf]

Supplemental Material

Eruptive Seborrheic Keratoses are Associated with a Co-occurring Malignancy in the Majority of Reported Cases: A Systematic Review

Sara Mirali (PhD)[1], Asfandyar Mufti (MD)[2], Rafael Paolo Lansang (BHSc)[3], Muskaan Sachdeva (BHSc)[1], Jensen Yeung (MD, FRCPC)[2]

[1]Faculty of Medicine, University of Toronto, Canada

[2]Department of Dermatology, University of Toronto, Toronto, Canada

[3]Faculty of Health Sciences, McMaster University, Hamilton, Canada

Supplemental File 1: Search strategy and outcome measures

#	Searches	Results
1	Leser-Trelat.mp. [mp=ti, ab, ot, nm, hw, fx, kf, ox, px, rx, ui, an, sy, tn, dm, mf, dv, kw, dq]	462
2	Stucco keratosis.mp. [mp=ti, ab, ot, nm, hw, fx, kf, ox, px, rx, ui, an, sy, tn, dm, mf, dv, kw, dq]	43
3	Stucco keratoses.mp. [mp=ti, ab, ot, nm, hw, fx, kf, ox, px, rx, ui, an, sy, tn, dm, mf, dv, kw, dq]	14
4	Large cell acanthoma.mp. [mp=ti, ab, ot, nm, hw, fx, kf, ox, px, rx, ui, an, sy, tn, dm, mf, dv, kw, dq]	63
5	Dermatosis papulosa nigra.mp. [mp=ti, ab, ot, nm, hw, fx, kf, ox, px, rx, ui, an, sy, tn, dm, mf, dv, kw, dq]	168
6	Melanoacanthoma.mp. [mp=ti, ab, ot, nm, hw, fx, kf, ox, px, rx, ui, an, sy, tn, dm, mf, dv, kw, dq]	191
7	Borst Jadassohn.mp. [mp=ti, ab, ot, nm, hw, fx, kf, ox, px, rx, ui, an, sy, tn, dm, mf, dv, kw, dq]	66
8	Inverted follicular keratosis.mp. [mp=ti, ab, ot, nm, hw, fx, kf, ox, px, rx, ui, an, sy, tn, dm, mf, dv, kw, dq]	173
9	Inverted follicular keratoses.mp. [mp=ti, ab, ot, nm, hw, fx, kf, ox, px, rx, ui, an, sy, tn, dm, mf, dv, kw, dq]	21
10	Tumor of the follicular infundibulum.mp. [mp=ti, ab, ot, nm, hw, fx, kf, ox, px, rx, ui, an, sy, tn, dm, mf, dv, kw, dq]	93
11	Lichenoid keratosis.mp. [mp=ti, ab, ot, nm, hw, fx, kf, ox, px, rx, ui, an, sy, tn, dm, mf, dv, kw, dq]	200
12	Lichenoid keratoses.mp. [mp=ti, ab, ot, nm, hw, fx, kf, ox, px, rx, ui, an, sy, tn, dm, mf, dv, kw, dq]	25
13	Seborrheic keratosis.mp. [mp=ti, ab, ot, nm, hw, fx, kf, ox, px, rx, ui, an, sy, tn, dm, mf, dv, kw, dq]	4218
14	Seborrheic keratoses.mp. [mp=ti, ab, ot, nm, hw, fx, kf, ox, px, rx, ui, an, sy, tn, dm, mf, dv, kw, dq]	1153
15	13 or 14	4689
16	Irritated.mp. [mp=ti, ab, ot, nm, hw, fx, kf, ox, px, rx, ui, an, sy, tn, dm, mf, dv, kw, dq]	2985
17	Inflamed.mp. [mp=ti, ab, ot, nm, hw, fx, kf, ox, px, rx, ui, an, sy, tn, dm, mf, dv, kw, dq]	58549
18	Hyperkeratotic.mp. [mp=ti, ab, ot, nm, hw, fx, kf, ox, px, rx, ui, an, sy, tn, dm, mf, dv, kw, dq]	5787
19	Acanthotic.mp. [mp=ti, ab, ot, nm, hw, fx, kf, ox, px, rx, ui, an, sy, tn, dm, mf, dv, kw, dq]	832

20	Adenoid.mp. [mp=ti, ab, ot, nm, hw, fx, kf, ox, px, rx, ui, an, sy, tn, dm, mf, dv, kw, dq]	26753
21	Reticulated.mp. [mp=ti, ab, ot, nm, hw, fx, kf, ox, px, rx, ui, an, sy, tn, dm, mf, dv, kw, dq]	4756
22	Macular.mp. [mp=ti, ab, ot, nm, hw, fx, kf, ox, px, rx, ui, an, sy, tn, dm, mf, dv, kw, dq]	133472
23	Clonal.mp. [mp=ti, ab, ot, nm, hw, fx, kf, ox, px, rx, ui, an, sy, tn, dm, mf, dv, kw, dq]	146574
24	16 or 17 or 18 or 19 or 20 or 21 or 22 or 23.mp.	378670
25	15 and 24.mp. [mp=ti, ab, ot, nm, hw, fx, kf, ox, px, rx, ui, an, sy, tn, dm, mf, dv, kw, dq]	457
26	Eruptive.mp. [mp=ti, ab, ot, nm, hw, fx, kf, ox, px, rx, ui, an, sy, tn, dm, mf, dv, kw, dq]	6038
27	Abrupt.mp. [mp=ti, ab, ot, nm, hw, fx, kf, ox, px, rx, ui, an, sy, tn, dm, mf, dv, kw, dq]	48354
28	Explosive.mp. [mp=ti, ab, ot, nm, hw, fx, kf, ox, px, rx, ui, an, sy, tn, dm, mf, dv, kw, dq]	26307
29	Sudden.mp. [mp=ti, ab, ot, nm, hw, fx, kf, ox, px, rx, ui, an, sy, tn, dm, mf, dv, kw, dq]	267539
30	2 or 3 or 4 or 5 or 6 or 7 or 8 or 9 or 10 or 11 or 12 or 13 or 14 or 25	5464
31	26 or 27 or 28 or 29	345027
32	30 and 31	235
33	1 or 32	558

Database(s): **Ovid MEDLINE: Epub Ahead of Print, In-Process & Other Non-Indexed Citations, Ovid MEDLINE® Daily and Ovid MEDLINE®** 1946-Present**, Embase Classic+Embase** 1947 to 2020 July 26

Outcomes Measures

1. "Patient self-report?" was marked "yes" if the ESK latency period was self-described by the patient.
2. "Latency period" was defined as the time between identification of ESK and diagnosis of the coinciding condition. Identification of ESK preceded the diagnosis of the coinciding condition.

3. "Clinical improvement of the associated condition" was noted as "yes" if there was resolution (i.e. tumor regression) of the disease that co-occurred with ESK.
4. Correlation between treatment of associated condition and ESK improvement was noted as "yes" if there was resolution of ESK following treatment of the coinciding condition.
5. "ESK resolution period" was defined as the period of time between treatment of the coinciding condition and improvement of the ESK lesions.
6. "Recovery of ESK" was defined as "partial" if there was a moderate improvement in the ESK lesions and "complete" if there were no ESK lesions present.
7. "Associated treatment stopped" was noted as "yes" if the patient stopped taking the coinciding medication.
8. "Correlation between stopping treatment and ESK improvement" was noted as "yes" if there was resolution of ESK following treatment cessation.
9. "Naranjo ADR Scale" refers to the probability that the drug caused ESK as per the Naranjo scale.[1]

References

1. Naranjo CA, Busto U, Sellers EM, et al. A method for estimating the probability of adverse drug reactions. *Clin Pharmacol Ther*. Aug 1981;30(2):239-45. doi:10.1038/clpt.1981.154

Figure S1: Literature screening flow diagram using the Preferred Reporting Items for Systematic Reviews and Meta-Analyses (PRSMA) guidelines. Figure adapted from http://prisma-statement.org.

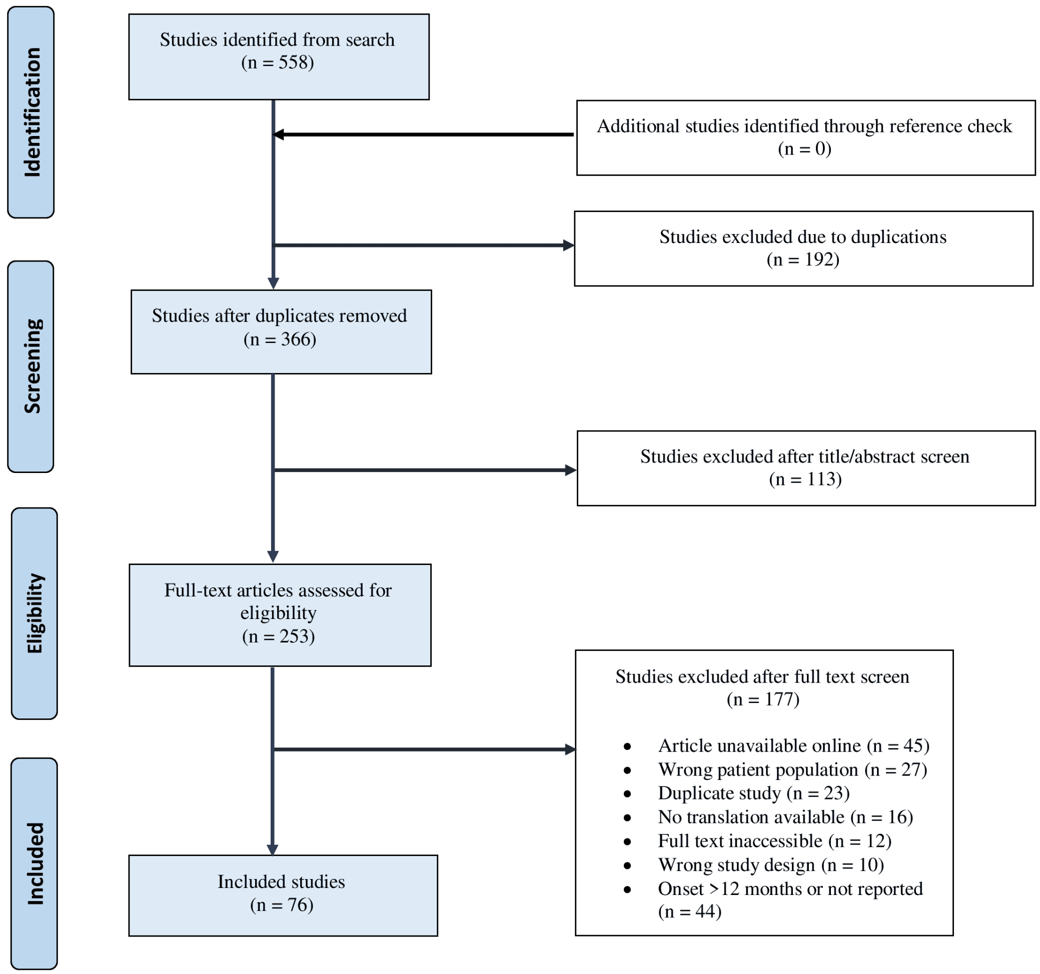

Table S1. Quality appraisal of case reports and case series studies

Study Design	Does the patient(s) represent(s) the whole experience of the investigator (centre) or is the selection method unclear to the extent that other patients with similar presentation may not have been reported?	Was the exposure adequately ascertained?	Was the outcome adequately ascertained?	Were other alternative causes that may explain the observation ruled out?	Was there a challenge/rechallenge phenomenon?	Was there a dose–response effect?	Was follow-up long enough for outcomes to occur?	Is the case(s) described with sufficient details to allow other investigators to replicate the research or to allow practitioners to make inferences related to their own practice?	Overall Rating
CR[1]	Unclear	Y	Y	N	N	N	N	N	Poor
CR[2]	Unclear	Y	Y	N	N	NA	N	N	Poor
CR[3]	Unclear	Y	Y	N	N	NA	N	N	Poor
CR[4]	Unclear	Y	N	N	N	NA	N	N	Poor
CR[5]	Unclear	N	Y	N	N	NA	Y	N	Poor
CR[6]	Unclear	N	Y	N	N	NA	Y	N	Poor
CR[7]	Unclear	N	Y	N	N	NA	N	N	Poor
CR[8]	Unclear	N	Y	N	N	NA	N	N	Poor
CR[9]	Unclear	Y	Y	N	N	NA	N	N	Poor
CR[10]	Unclear	N	Y	N	N	NA	N	N	Poor
CR[11]	Unclear	N	Y	N	N	NA	N	N	Poor
CR[12]	Unclear	Y	Y	N	N	NA	N	N	Poor
CR[13]	Unclear	Y	Y	N	N	NA	N	N	Poor
CR[14]	Unclear	Y	Y	N	N	NA	N	N	Poor
CR[15]	Unclear	N	Y	N	N	NA	N	Y	Poor
CR[16]	Unclear	Y	Y	N	N	NA	Y	Y	Fair
CR[17]	Unclear	Y	Y	N	N	NA	Y	Y	Fair
CR[18]	Unclear	N	Y	Y	N	NA	Y	Y	Fair
CR[19]	Y	Y	Y	N	N	NA	N	N	Fair
CR[20]	Y	Y	Y	Y	N	NA	N	N	Fair
CS[21]	Y	Y	Y	N	N	N	Y	N	Fair
CR[22]	Unclear	Y	Y	N	N	N	Y	Y	Fair
CS[23]	Y	N	Y	Y	N	N	Y	Y	Fair
CR[24]	Unclear	Y	Y	N	N	NA	N	Y	Fair
CR[25]	Y	Y	Y	N	N	NA	N	Y	Fair
CR[26]	Unclear	Y	Y	N	N	NA	N	Y	Fair
CR[27]	Unclear	Y	Y	N	N	NA	N	Y	Fair
CR[28]	Y	Y	Y	N	N	NA	N	Y	Fair
CR[29]	Y	Y	N	N	N	NA	Y	Y	Fair
CR[30]	Y	Y	Y	Y	N	NA	N	Y	Fair
CR[31]	Unclear	Y	Y	Y	N	NA	N	Y	Fair

CR[32]	Y	Y	Y	N	N	NA	Y	N	Fair
CR[33]	Unclear	Y	Y	N	N	NA	N	Y	Fair
CR[34]	Unclear	Y	Y	N	N	NA	Y	Y	Fair
CR[35]	Y	Y	Y	N	N	NA	N	Y	Fair
CR[36]	Y	Y	Y	Y	N	NA	N	Y	Fair
CR[37]	Unclear	Y	Y	N	N	NA	Y	Y	Fair
CR[38]	Unclear	Y	Y	N	N	NA	Y	Y	Fair
CR[39]	Unclear	Y	Y	N	N	NA	Y	Y	Fair
CR[40]	Unclear	Y	Y	N	N	NA	Y	N	Fair
CR[41]	Unclear	Y	Y	N	N	NA	Y	Y	Fair
CR[42]	Unclear	Y	Y	N	N	NA	Y	N	Fair
CR[43]	Unclear	Y	Y	N	N	NA	Y	N	Fair
CR[44]	Unclear	Y	Y	N	N	NA	N	Y	Fair
CR[45]	Unclear	N	Y	Y	N	NA	Y	Y	Fair
CR[46]	Unclear	N	Y	Y	N	NA	Y	Y	Fair
CR[47]	N	Y	Y	N	NA	N	Y	N	Fair
CR[48]	Unclear	Y	Y	Y	N	N	Y	Y	Good
CR[49]	Y	Y	Y	Y	N	Y	Y	Y	Good
CR[50]	Y	Y	Y	N	N	Y	Y	Y	Good
CR[51]	Y	Y	Y	N	N	Y	Y	Y	Good
CR[52]	Unclear	Y	Y	Y	Y	NA	N	Y	Good
CR[53]	Unclear	Y	Y	Y	Y	NA	Y	Y	Good
CR[54]	Y	Y	Y	Y	N	NA	Y	Y	Good
CR[55]	Y	Y	Y	Y	N	NA	Y	Y	Good
CR[56]	Y	Y	Y	Y	N	NA	N	Y	Good
CR[57]	Y	Y	Y	Y	N	NA	Y	Y	Good
CR[58]	Unclear	Y	Y	Y	N	NA	Y	Y	Good
CR[59]	Unclear	Y	Y	Y	N	NA	Y	Y	Good
CR[60]	Unclear	Y	Y	Y	N	NA	Y	Y	Good
CR[61]	Unclear	Y	Y	Y	N	NA	Y	Y	Good
CR[62]	Y	Y	Y	N	Y	NA	Y	Y	Good
CR[63]	Unclear	Y	Y	Y	N	NA	Y	Y	Good
CR[64]	Y	Y	Y	Y	N	NA	Y	Y	Good
CR[65]	Unclear	Y	Y	Y	N	NA	Y	Y	Good
CS[66]	Unclear	Y	Y	N	Y	NA	Y	Y	Good
CR[67]	Unclear	Y	Y	Y	N	NA	Y	Y	Good
CR[68]	Unclear	Y	Y	Y	N	NA	Y	Y	Good
CS[69]	Y	Y	Y	N	N	NA	Y	Y	Good
CR[70]	Y	Y	Y	N	N	NA	Y	Y	Good
CR[71]	Unclear	Y	Y	Y	N	NA	Y	Y	Good
CR[72]	Unclear	Y	Y	Y	Y	NA	Y	Y	Good
CR[73]	Y	Y	Y	Y	N	NA	N	Y	Good

Table S2. Quality appraisal of case control studies

		Study		
		Lindelof[1]	Insuasty[2]	Fink[3]
Is the case definition adequate?	Yes, with independent validation (1 point)	1	1	1
Representativeness of the cases	Consecutive or obviously representative series of cases (1 point)	1	1	1
Selection of Controls	Community controls (1 point)	1	0	0
Definition of Controls	No history of disease (1 point)	0	0	0
Comparability of cases and controls on the basis of the design or analysis	Study controls for most important factor (1 point) and for any additional factor (2 points)	2	2	2
Ascertainment of exposure	Secure record (1 point)	1	1	1
Same method of ascertainment for cases and controls	Yes (1 point)	1	1	1
Non-response rate	Same rate for both groups	1	0	1
Total Score (maximum = 9)		8	6	7

References

1. Lindelof B, Sigurgeirsson B, Melander S. Seborrheic keratoses and cancer. Jun 1992;1(6):947-50.
2. Insuasty J, Diaz L, Berbeo M, Ballesteros Z, Mantilla A. Eruptive seborrheic keratosis (ESK) and its association with gastrointestinal cancer (GIC): A case-control study and meta-analysis. 2016 (Supplement 2):ii95-ii96.
3. Fink AM, Filz D, Krajnik G, Jurecka W, Ludwig H, Steiner A. Seborrhoeic keratoses in patients with internal malignancies: a case-control study with prospective accrual of patients. Nov 2009;1(11):1316-9.

Table S3: Summary of Demographic Information and Outcomes of Patients with ESK and a Malignant or Non-malignant/No Disease

Demographics	Malignant	Non-malignant/ No disease
**Sex**		
Female	29/58 (50.0%)	2/8 (25.0%)
Male	29/58 (50.0%)	6/8 (75.0%)
NR	12	5
Total	70	13
**Age**		
Mean Age ± std (years)	62.8 ± 14.2	44.9 ± 23.1
Age Range (years)	20 - 92	13 - 74
NR	11	5
**SK outcome measure**		
Visual appearance	42/69 (60.9%)	6/11 (54.5%)
Visual appearance, histology	27/69 (39.1%)	5/11 (45.5%)
NR	1	2
**Latency**		
Mean ± std (months)	4.0 ± 2.3	3.1 ± 2.7
Range (months)	0.25 - 9	0.75 - 6
NR	16	6
**Correlation between treatment of associated condition and ESK improvement**		
Yes	19/29 (65.5)	1/2 (50.0)
No	10/29 (34.4)	1/2 (50.0)
NR	41	11
**Resolution**		
None	0/19 (0.0%)	1/2 (50.0%)
Partial	11/19 (57.9%)	1/2 (50.0%)
Complete	8/19 (42.1%)	0/2 (0.0%)
NR	51	11
**Resolution Period**		
Mean ± std (months)	4.6 ± 4.2	6.0
Range (months)	0.25 - 12	6.0
NR	62	12

Abbreviations: ESK eruptive seborrheic keratoses; **NR** not reported; **std** standard deviation.

Table S4. Summary of Included Cases of ESK and Associated Conditions

Study Information		Demographic Information			ESK Latency		Treatment of Associated Condition and ESK Resolution					
Study Design, Level of Evidence	Sample size	Age, Sex	Associated Condition(s)	Comorbidities	Patient self-report?	Latency period (months)	Treatment for Associated Condition(s)	Clinical Improvement of Associated Condition	Correlation Between Treatment of Associated Condition and ESK Improvement	ESK Resolution Period (months)	Recovery of ESK
CR[1], 5	1	72, M	acute leukemia	NR	N	0.25	chemotherapy	No	NR	NR	NR
CR[2], 5	1	43, M	B-cell lymphoma	irritable bowel syndrome, depression, sleep apnea	N	6	excision	Yes	Yes	1	partial
CR[3], 5	1	59, F	lymphocytic lymphoma, adenocarcinoma of the large bowel	obesity	Y	2	chemotherapy	NR	No	NR	NR
CR[4], 5	1	20, M	relapsed pre-B-cell acute lymphocytic leukemia	NR	Y	1	chemotherapy	NR	NR	NR	NR
CR[5], 5	1	57, M	Sézary syndrome	NR	N	5	interferon alfa, extracorporeal photophoresis	Yes	Yes	6	complete
CR[6], 5	1	55, M	Sézary syndrome	NR	Y	4	NR	NR	NR	NR	NR
CR[7], 5	1	74, M	Sézary syndrome	DM	Y	3	oral prednisolone	Yes	Yes	0.75	partial
CR[8], 5	1	63, M	Sezary syndrome, transitional cell carcinoma of the bladder	NR	N	1	photochemotherapy, chemotherapy	NR	Yes	2	complete
CR[9], 5	1	58, M	Sézary syndrome, lymphoma transformation	NR	N	7	electron beam irradiation, chemotherapy	NR	Yes	NR	complete
CR[10], 5	1	76, F	mycosis fungoides	NR	N	4	extracorporeal photopheresis	NR	NR	NR	NR
CR[11], 5	1	35, F	mycosis fungoides	NR	Y	Within weeks	topical steroid, photocheotherapy, etretinate, and intravenous interleukin-1	Yes	No	NR	NR
CR[12], 5	1	71, M	mycosis fungoides	NR	Y	3	NR	NR	NR	NR	NR
CR[13], 5	1	61, F	mycosis fungoides	NR	Y	0.75	photochemotherapy	Yes	Yes	NR	complete
CR[14], 5	1	60, M	relapsed mycosis fungoides	NR	Y	9	NR	NR	NR	NR	NR

CR[15], 5	1	45, F	adenocarcinoma of the ampulla of Vater	NR	Y	6	surgery	Yes	No	NR	NR
CR[16], 5	1	70, F	adenocarcinoma of the bile duct	ulcerative colitis	N	3	NR	NR	NR	NR	NR
CR[17], 5	1	57, M	metastisized adenocarcinoma of the gallbladder	immunosuppr essive (previous renal transplant)	Y	9	palliative care	NR	NR	NR	NR
CR[18], 5	1	68, M	adenocarcinoma of the prostate	NR	Y	2	surgery	NR	No	NR	NR
CR[19], 5	1	69, M	adenocarcinoma of the stomach	NR	Y	2	NR	NR	NR	NR	NR
CR[20], 5	1	52, F	clear cell adenocarcinoma of left ovary	NR	Y	6	chemotherapy, surgery	Yes	Yes	NR	complete
CR[21], 5	1	61, F	endometrial adenocarcinoma	depression	Y	1	surgery	Yes	Yes	NR	complete
CR[22], 5	1	57, F	endometrial adenocarcinoma	obesity	Unclea r	Within weeks	surgery	Yes	NR	NR	NR
CR[23], 5	1	69, M	gastric adenocarcinoma	obesity, heart disease	Y	6	chemotherapy	Yes	Yes	NR	partial
CR[24], 5	1	41, F	gastric adenocarcinoma	NR	Unclea r	6	NR	NR	NR	NR	NR
CR[25], 5	1	56, M	metastatic adenocarcinoma	obesity	Y	3	chemotherapy	No	NR	NR	NR
CR[26], 5	1	76, F	ovarian adenocarcinoma, moderate cervical dysplasia	NR	Y	Within weeks	surgery	NR	No	NR	NR
CR[27], 5	1	92, M	pancreatic adenocarcinoma	NR	Y	2	palliative care	NR	NR	NR	NR
CR[28], 5	1	54, M	prostate adenocarcinoma	refractory cutaneous lupus erythematosu s	N	3	surgery	NR	Yes	1	partial
CR[29], 5	1	54, F	visceral adenocarcinoma	recent hysterectomy for uterine	Y	1	surgery	NR	NR	NR	NR

				adenocarcinoma							
CR[30], 5	1	75, M	basal cell carcinoma of the lower eyelid	NR	Y	3	excision	NR	NR	NR	NR
CR[31], 5	1	67, M	bladder carcinoma	COPD	Unclear	4	surgery, immunotherapy	NR	Yes	9	partial
CR[32], 5	1	69, F	cholangiocarcinoma	NR	Y	9	none	NR	NR	NR	NR
CR[33], 5	1	75, M	nasopharyngeal carcinoma	NR	Y	3	radiation	NR	NR	NR	NR
CR[34], 5	1	78, F	fallopian tube carcinoma, endometrioid adenocarcinoma	DM type I, hyperlipidemia, hypothyroidism	Y	0.5	NR	NR	NR	NR	NR
CR[35], 5	1	57, M	hepatocellular carcinoma	NR	Y	6	hepatic arterial embolization	No	NR	NR	NR
CR[36], 5	1	48, F	intraductal carcinoma of right breast	NR	Y	3	NR	NR	NR	NR	NR
CR[37], 5	1	65, M	invasive squamous cell carcinoma	NR	Y	2	surgery, radiation, chemotherapy	Yes	No	NR	NR
CR[38], 5	1	53, M	pancreatic carcinoma	NR	Y	6	surgery	No	Yes	NR	partial
CR[39], 5	1	70, F	Klatskin's cholangiocarcinoma	NR	Unclear	3	palliative care	NR	NR	NR	NR
CR[40], 5	1	68, F	renal cell carcinoma	NR	Y	1	NR	NR	NR	NR	NR
CR[41], 5	1	69, M	squamous cell carcinoma	NR	Y	2	surgery	Yes	Yes	NR	partial
CR[42], 5	1	85, F	squamous cell carcinoma of the lung	NR	Y	6	NR	NR	NR	NR	NR
CR[43], 5	1	NR, NR	relapsed squamous cell carcinoma of larynx	NR	Y	1	chemotherapy, radiation	NR	No	NR	NR
CR[44], 5	1	64, F	thymic carcinoma	hypertension	Y	6	Unspecified treatment	No	NR	NR	NR
CR[45], 5	1	71, F	metastasized malignant melanoma	DM type 2, hypertension, heart disease	Y	3	NR	NR	NR	NR	NR
CR[46], 5	1	54, M	melanoma	hypertension	Y	3	excision	Yes	Yes	5	partial
CS[47], 4	2	72, M	carcinoma of the transverse colon	NR	Y	3	surgery	No	NR	NR	NR
		67, F	carcinoma of the rectum	NR	Y	6	radiation	Yes	Yes	NR	partial

CR[48], 5	1	66, F	hepatic haemangiopericytoma	hypoglycemia	Unclear	6	vessel embolization	Yes	Yes	NR	complete
CR[49], 5	1	41, F	malignant hemangiopericytoma	hypoglycemia	Y	6	surgery	Yes	Yes	12	complete
CR[50], 5	1	72, M	porphyria cutanea tarda and malignant hepatoma	NR	Y	6	NR	NR	NR	NR	NR
CR[51], 5	1	79, F	uterine leiomyosarcoma	hypertension	Y	6	surgery	Yes	No	NR	NR
CR[52], 5	1	71, F	breast cancer	obesity	Y	Within weeks	NR	NR	NR	NR	NR
CS[53], 4	2	41, F	infiltrating ductal carcinoma of the breast	NR	Y	6	surgery, radiation	NR	No	NR	NR
		74, F	infiltrating ductal carcinoma of the breast	recurrent diverticulitis, esophageal diverticulum		3	surgery, radiation	NR	No	NR	NR
CR[54], 5	1	90, F	tumor of the ampulla of Vater	NR	Unclear	Within weeks	endoscopic stent	NR	NR	NR	NR
CR[55], 5	1	29, M	recurrent anaplastic ependymomas	NR	Unclear	3	chemotherapy, radiation	No	NR	NR	NR
CR[56], 5	1	81, M	retroperitoneal solitary fibrous tumor	primary hypothyroidism, hypoglycemia	Y	6	surgery, palliative care	Yes	Yes	NR	partial
CR[57], 5	1	66, F	renal cell carcinoma	DM, hypertension, heart disease	Y	7	surgery	Yes	Yes	NR	partial
CR[58], 5	1	74, M	erythrodermic pityriasis rubra pilaris	NR	Y	1	methotrexate and acitretin	Yes	Yes	6	NR
CR[59], 5	1	71, M	syphillis	NR	Unclear	6	NR	NR	NR	NR	NR
CR[60], 5	1	32, M	HIV	NR	Y	0.75	nevirapine, stavudine and lamivudine	NR	NR	NR	NR
CR[61], 5	1	22, M	DM type 2	obesity, hypertension, sleep apnea, depression	Y	6	metformin	No	No	NR	none
CR[62], 5	1	66, F	NR	NR	Y	"Less than 1 year"	NR	NR	NR	NR	NR

CR[63], 5	1	37, F	NR	NR	Y	1	NR	NR	NR	NR	NR
CR[64], 5	1	44, M	NR	NR	Y	1	NR	NR	NR	NR	partial
CR[65], 5	1	13, M	NR	obesity	Unclear	6	NR	NR	NR	NR	NR

Abbreviations: COPD chronic obstructive pulmonary disease; **CR** case report; **CS** case series; **DM** diabetes mellitus; **ESK** eruptive seborrheic keratoses; **F** female; **HIV** human immunodeficiency virus; **M** male; **NR** not reported

References

1. Greer KE, Hawkins H, Hess C. Leser-Trelat associated with acute leukemia. Oct 1978;1(10):1552.
2. Hu S, Granter SR, Haynes HA, Miller DM. Skin spicules: A newly described paraneoplastic phenomenon associated with a marginal zone B-cell lymphoma. May 2009;1(5):852-5.
3. Wagner RF, Wagner KD. Malignant neoplasms and the Leser-Trelat sign. Sep 1981;1(9):598-9.
4. Fasoldt JJ, Brumwell ER, Lackey JN. Leser-Trelat sign presenting in a patient with recurrent pre-B-cell acute lymphocytic leukemia. Jan 2012;1(1):33-5.
5. Cohen JH, Lessin SR, Vowels BR, Benoit B, Witmer WK, Rook AH. The sign of Leser-Trelat in association with Sezary syndrome: Simultaneous disappearance of seborrheic keratoses and malignant T-cell clone during combined therapy with photopheresis and interferon alfa [9]. 1993;(9):1213-1215.
6. Dantzig PI. Sign of Leser-Trelat. Nov 1973;1(5):700-1.
7. Ikari Y, Ohkura M, Morita M, Seki K, Kubota Y, Mizoguchi M. Leser-Trelat sign associated with Sezary syndrome. Jan 1995;1(1):62-7.
8. Martinez-Moran C, Sanz-Munoz C, Miranda-Romero A. [Leser-Trelat sign associated with Sezary syndrome and transitional cell carcinoma of the bladder]. [Spanish]. Apr 2007;1(3):214-5.
9. Wieselthier JS, Bhawan J, Koh HK. Transformation of Sezary syndrome and the sign of Leser-Trelat: a histopathologic study. Sep 1990;1(3 Pt 1):520-2.
10. McCrary ML, Davis LS. Sign of Leser-Trelat and mycosis fungoides. Apr 1998;1(4):644.
11. Miyako F, Dekio S, Tamura H, et al. Mycosis fungoides with Leser-Trelat sign: the first report of a patient from Japan. Mar 1994;1(3):189-93.
12. Rowe B, Shevchenko A, Yosipovitch G. Leser-Trelat Sign in Tumor-Stage Mycosis Fungoides. Apr 18 2016;1(4)
13. Toonstra J, Ramselaar CG, van der Putte SC. Leser-Trelat sign in mycosis fungoides. A further case report. 1985;1(4):247-9.

14. Alsaif F, Alkhayal FA, Aldahash R, Alhumaidi A. Leser-Trelat Sign Presenting in a Patient with Relapsing Mycosis Fungoides. May 2018 (2):436-441.
15. Klimopoulos S, Kounoudes C, Pantelidaki C, Skrepetou K, Papoudos M, Katsoulis H. The leser-trelat sign in association with carcinoma of the ampulla of Vater. May 2001;1(5):1623-6.
16. Lee CH, Clark AR, Thorpe ME, Mackie BS, Firkin FC. Bile duct adenocarcinoma with a Leser-Trelat sign and pure red blood cell aplasia. 1980;(7):1657-1660.
17. Kocyigit P, Akay BN, Arica E, Anadolu RY, Erdem C. Post-renal transplantation Leser-Trelat sign associated with carcinoma of the gallbladder: a rare association. Jun 2007;1(6):779-81.
18. Kluger N, Guillot B. Sign of Leser-Trelat with an adenocarcinoma of the prostate: A case report. 2009;(8):no pagination.
19. Sperry K, Wall J. Adenocarcinoma of the stomach with eruptive seborrheic keratoses. The sign of Leser-Trelat. 1980;(9):2434-2437.
20. Kebria MM, Belinson J, Kim R, Mekhail TM. Malignant acanthosis nigricans, tripe palms and the sign of Leser-Tre'lat, a hint to the diagnosis of early stage ovarian cancer: a case report and review of the literature. May 2006;1(2):353-5.
21. Storer M, Duncan LM, Kourosh AS. Eruptive inflamed seborrheic keratoses in the setting of endometrial adenocarcinoma.155-156.
22. Aylesworth R, Vance JC. Multiple hamartoma syndrome with endometrial carcinoma and the sign of Leser-Trelat. Feb 1982;1(2):136-8.
23. Yeh JS, Munn SE, Plunkett TA, Harper PG, Hopster DJ, du Vivier AW. Coexistence of acanthosis nigricans and the sign of Leser-Trelat in a patient with gastric adenocarcinoma: a case report and literature review. [Review] [50 refs]. Feb 2000;1(2 Pt 2):357-62.
24. Zhang N, Qian Y, Feng AP. Acanthosis nigricans, tripe palms, and sign of Leser-Trelat in a patient with gastric adenocarcinoma: case report and literature review in China. [Review]. Mar 2015;1(3):338-42.
25. Jacobs MI, Rigel DS. Acanthosis nigricans and the sign of Leser-Trelat associated with adenocarcinoma of the gallbladder. 1981;(2):325-328.
26. Holguin T, Padilla RS, Ampuero F. Ovarian adenocarcinoma presenting with the sign of Leser-Trelat. Sep 1986;1(1):128-32.
27. Cuervo Pinna MA. [Leser-Trelat sign associated with pancreatic cancer]. [Spanish]. May 20 2016;1(10):470.
28. da Rosa AC, Pinto GM, Bortoluzzi JS, Duquia RP, de Almeida HL, Jr. Three simultaneous paraneoplastic manifestations (ichthyosis acquisita, Bazex syndrome, and Leser-Trelat sign) with prostate adenocarcinoma. Sep 2009;1(3):538-40.
29. Ronchese F. KERATOSES, CANCER and 'THE SIGN of LESER-TRELAT'. 1965;(8):1003-1006.
30. Garg R, Madan S, Prakash P, Chander R, Choudhary M. Leser-Trelat Syndrome in a Male with Breast Carcinoma and Eyelid Basal Cell Carcinoma. Apr 2018 (3):161-164.
31. Stollmeier A, Rosario BA, Mayer BL, Frandoloso GA, Magalhaes FLGM, Marques GL. Seborrheic Keratoses as the First Sign of Bladder Carcinoma: Case Report of Leser-Trelat Sign in a Rare Association with Urinary Tract Cancer. 2016:no pagination.

32. Scully C, Barrett WA, Gilkes J, Rees M, Sarner M, Southcott RJ. Oral acanthosis nigricans, the sign of Leser-Trelat and cholangiocarcinoma. Sep 2001;1(3):506-7.
33. Li M, Yang LJ, Zhu XH, et al. The Leser-Trelat sign is associated with nasopharyngeal carcinoma: case report and review of cases reported in China. [Review] [10 refs]. Jan 2009;1(1):52-4.
34. West L, Carlson M, Wallis L, Goff HW. The Sign of Leser-Trelat and Malignant Acanthosis Nigricans Associated With Fallopian Tube Carcinoma. 11 2018;1(5):1116-1119.
35. Tajima H, Mitsuoka S, Ohtsuka E, et al. A case of hepatocellular carcinoma with the sign of Leser-Trelat: a possible role of a cutaneous marker for internal malignancy. Jan-Feb 1991;(1):53-56.
36. Shamsadini S, Wadji MB, Shamsadini A. Surrounding ipsilateral eruptive seborrheic keratosis as a warning sign of intraductal breast carcinoma and Paget's disease (Leser Trelat sign). Oct 31 2006;1(6):27.
37. Gaduputi V, Chandrala C, Tariq H, Kanneganti K. Sign of Leser-Trelat associated with esophageal squamous cell cancer. 2014:no pagination.
38. Ohashi N, Hidaka N. Pancreatic carcinoma associated with the Leser-Trelat sign. 1997;(2):155-160.
39. Morgenthau A, Almudaires A. Klatskin's cholangiocarcinoma presenting with the sign of Leser-Trelat. Oct 30 2019;1(10)
40. Barth D, Puhlmann S, Barth J. A Case of Leser-Trelat Syndrome Associated with a Renal Cell Carcinoma. Dec 2015 (3):245-248.
41. Doll DC, McCagh MF, Welton WA. Sign of Leser-Trelat. Jul 18 1977;1(3):236-7.
42. Sardon C, Dempsey T. The Leser-Trelat sign. Dec 2017;1(12):918.
43. Nyati A, Kalwaniya S, Jain S, Soni B. Sign of Leser-Trelat in association with laryngeal carcinoma. Jan-Feb 2016;1(1):112.
44. Mendes GB, Zanetti G, Marchiori E. Leser-Trelat Sign Secondary to Thymic Carcinoma. May 2018;1(5):286-287.
45. Pereira R, Carvalho SD, Cruz JF. Leser-Trelat sign in metastatic melanoma to pleura. Apr 03 2019;1(4)
46. Ellis DL, Kafka SP, Chow JC, et al. Melanoma, growth factors, acanthosis nigricans, the sign of leser-trelat, and multiple acrochordons. A possible role for alpha-transforming growth factor in cutaneous paraneoplastic syndromes. 1987;(25):1582-1587.
47. Liddell K, White JE, Caldwell IW. Seborrhoeic keratoses and carcinoma of the large bowel. Three cases exhibiting the sign of Lester-trélat. *Br J Dermatol*. Apr 1975;92(4):449-52. doi:10.1111/j.1365-2133.1975.tb03107.x
48. da Costa Franca AF, Siqueira NS, Carvalheira JB, Saad MJ, Souza EM. Acanthosis nigricans, tripe palms and the sign of Leser-Trelat in a patient with a benign hepatic neoplasia. Jul 2007;1(6):846-8.
49. Mayou SC, Benn JJ, Sonksen PH, Black MM. Paraneoplastic rhinophyma and the Leser-Trelat sign. May 1989;1(3):253-5.
50. Harrington CI. Letter: Leser-Trelat sign with porphyria cutanea tarda and malignant hepatoma. May 1976;1(5):730.
51. Abakka S, Elhalouat H, Khoummane N, et al. Uterine leiomyosarcoma and Leser-Trelat sign. Jan 05 2013;1(9860):88.
52. Al Ghazal P, Korber A, Klode J, Dissemond J. Leser-Trelat sign and breast cancer. May 11 2013;1(9878):1653.
53. Lynch HT, Fusaro RM, Pester JA, Lynch JF. Leser-Trelat sign in mother and daughter with breast cancer. Jun 1982;1(3):218-21.

54. Ho ML, Girardi PA, Williams D, Lord RV. Education and imaging. Gastrointestinal: The sign of Leser-Trelat. Apr 2008;1(4):672.
55. Hamada Y, Iwaki T, Muratani H, Imayama S, Fukui M, Tateishi J. Leser-Trelat sign with anaplastic ependymoma--an autopsy case. Jan 1997;1(1):97-100.
56. Mathez ALG, Moroto D, Dib SA, De Sa JR. Seborrheic keratoses and severe hypoinsulinemic hypoglycemia associated with insulin grow factor 2 secretion by a malignant solitary fibrous tumor. Apr 2016 (1):no pagination.
57. Fetil E, Ozkan S, Gurler N, Kusku E, Arda F, Gunes AT. Recurrent leser-trelat sign associated with two malignancies. 2002;1(3):254-5.
58. Gleeson CM, Chan I, Griffiths WAD, Bunker CB. Eruptive seborrhoeic keratoses associated with erythrodermic pityriasis rubra pilaris. 2009 (2):217-218.
59. Pimentel J, Wriston C, Bridges A. Secondary syphilis presenting as eruptive lichenoid keratoses. 2013 (5):e87-e88.
60. Inamadar AC, Palit A. Eruptive seborrhoeic keratosis in human immunodeficiency virus infection: a coincidence or 'the sign of Leser-Trelat'? *Br J Dermatol*. Aug 2003;149(2):435-6. doi:10.1046/j.1365-2133.2003.05463.x
61. Saraiya A, Al-Shoha A, Brodell RT. Hyperinsulinemia associated with acanthosis nigricans, finger pebbles, acrochordons, and the sign of Leser-Trelat. [Review]. May-Jun 2013;1(3):522-5.
62. Safa G, Darrieux L. Leser-Trelat sign without internal malignancy. 2011 (1):175-177.
63. Turan E, Yesilova Y, Yurt N, Kocarslan S. Leser-Trelat sign: does it really exist? 2013;1(2):123-7.
64. Turan E, Gurel MS, Erdemir AT. Leser-Trelat sign: a paraneoplastic process? :E14-5.
65. Hardy RD, Duvic M, Bleyer WA. The sign of Leser-Trelat. Mar 1997;1(3):234-7.

Table S5. Summary of Included Case Control Studies of ESK and Associated Conditions

Study Design	Sample size	Mean Age	Sex	Inclusion Criteria	Exclusion Criteria	Definition of ESK	SK Outcome Measure	ESK Diagnosis	Number of Patients with ESK (%)	Mean Latency Period (months)	Type of Malignancy	Number of Controls with ESK (%)	Main Findings
CC[1], 3b	150 cancer patients, 150 age and sex matched controls	Patients: 66.5; Control: 66.3	Both groups: 80F, 70M	(i) age > 45 years, (ii) patients: tumour diagnosed with cancer in the preceding 12 months, (iii) patients: no prior internal or cutaneous malignancies except basal cell carcinoma (iv) presence of SKs	NR	sudden development of >20 seborrhoeic keratoses within 6 months prior to the diagnosis of tumour	visual appearance	Physician	2 (1%)	NR	pancreatic adenocarcinoma (50%), esophageal squamous cell carcinoma (50%)	0 (0%)	ESK is rare, no association between ESK and malignancy
CC[2], 4	1752 cancer patients, 62 age and sex matched controls with multiple SK	Both groups: 63	Cancer patients: 1138F, 614M; Controls: NR	SK diagnosed between 1958-1983	NR	sudden eruption of >20 SK, patients reported latency period of 2-8 months	visual appearance	Investigator	6 (0.3%)	10.5	breast (33%), stomach (33%), pleura (17%), liver (17%)	5 (8%)	ESK is underdiagnosed but no association between ESK and malignancy
CC[3], 4	130 gastrointestinal tract/pancreatobiliary cancer patients, 260 controls	NR	NR	(i) subjects were at least 18 years old, (ii) patients with histopathological diagnosis of gastrointestinal tract/pancreatobiliary cancer, (iii) controls were hospitalized for any non-oncological cause	NR	>20 SKs within less than 6 months onset	visual appearance	NR	3 (2.3%)	NR	NR	0 (0%)	ESK is rare, sample size too small to determine association between ESK and malignancy

Abbreviations: CC case control; **ESK** eruptive seborrheic keratoses; **F** female; **M** male; **NR** not reported, **SK** seborrheic keratoses

References

1. Fink AM, Filz D, Krajnik G, Jurecka W, Ludwig H, Steiner A. Seborrhoeic keratoses in patients with internal malignancies: a case-control study with prospective accrual of patients. Nov 2009;1(11):1316-9.
2. Lindelof B, Sigurgeirsson B, Melander S. Seborrheic keratoses and cancer. 1992;(6):947-950.
3. Insuasty J, Diaz L, Berbeo M, Ballesteros Z, Mantilla A. Eruptive seborrheic keratosis (ESK) and its association with gastrointestinal cancer (GIC): A case-control study and meta-analysis. 2016 (Supplement 2):ii95-ii96.

PRISMA 2009 Checklist

Section/topic	#	Checklist item	Reported on page #
**TITLE**			1
Title	1	Identify the report as a systematic review, meta-analysis, or both.	Yes
**ABSTRACT**			2
Structured summary	2	Provide a structured summary including, as applicable: background; objectives; data sources; study eligibility criteria, participants, and interventions; study appraisal and synthesis methods; results; limitations; conclusions and implications of key findings; systematic review registration number.	Yes
**INTRODUCTION**			3
Rationale	3	Describe the rationale for the review in the context of what is already known.	Yes
Objectives	4	Provide an explicit statement of questions being addressed with reference to participants, interventions, comparisons, outcomes, and study design (PICOS).	Yes
**METHODS**			3-5
Protocol and registration	5	Indicate if a review protocol exists, if and where it can be accessed (e.g., Web address), and, if available, provide registration information including registration number.	The protocol was not registered on Prospero.
Eligibility criteria	6	Specify study characteristics (e.g., PICOS, length of follow-up) and report characteristics (e.g., years considered, language, publication status) used as criteria for eligibility, giving rationale.	Yes
Information sources	7	Describe all information sources (e.g., databases with dates of coverage, contact with study authors to identify additional studies) in the search and date last searched.	Yes
Search	8	Present full electronic search strategy for at least one database, including any limits used, such that it could be repeated.	Yes
Study selection	9	State the process for selecting studies (i.e., screening, eligibility, included in systematic review, and, if applicable, included in the meta-analysis).	Yes
Data collection process	10	Describe method of data extraction from reports (e.g., piloted forms, independently, in duplicate) and any processes for obtaining and confirming data from investigators.	Yes

Data items	11	List and define all variables for which data were sought (e.g., PICOS, funding sources) and any assumptions and simplifications made.	Yes
Risk of bias in individual studies	12	Describe methods used for assessing risk of bias of individual studies (including specification of whether this was done at the study or outcome level), and how this information is to be used in any data synthesis.	Yes
Summary measures	13	State the principal summary measures (e.g., risk ratio, difference in means).	Yes
Synthesis of results	14	Describe the methods of handling data and combining results of studies, if done, including measures of consistency (e.g., I^2) for each meta-analysis.	N/A

Section/topic	#	Checklist item	Reported on page #
Risk of bias across studies	15	Specify any assessment of risk of bias that may affect the cumulative evidence (e.g., publication bias, selective reporting within studies).	Yes
Additional analyses	16	Describe methods of additional analyses (e.g., sensitivity or subgroup analyses, meta-regression), if done, indicating which were pre-specified.	N/A
**RESULTS**			5-6
Study selection	17	Give numbers of studies screened, assessed for eligibility, and included in the review, with reasons for exclusions at each stage, ideally with a flow diagram.	Yes
Study characteristics	18	For each study, present characteristics for which data were extracted (e.g., study size, PICOS, follow-up period) and provide the citations.	Yes
Risk of bias within studies	19	Present data on risk of bias of each study and, if available, any outcome level assessment (see item 12).	Yes
Results of individual studies	20	For all outcomes considered (benefits or harms), present, for each study: (a) simple summary data for each intervention group (b) effect estimates and confidence intervals, ideally with a forest plot.	Yes (a only)
Synthesis of results	21	Present results of each meta-analysis done, including confidence intervals and measures of consistency.	N/A
Risk of bias across studies	22	Present results of any assessment of risk of bias across studies (see Item 15).	Yes
Additional analysis	23	Give results of additional analyses, if done (e.g., sensitivity or subgroup analyses, meta-regression [see Item 16]).	N/A
**DISCUSSION**			7-9
Summary of evidence	24	Summarize the main findings including the strength of evidence for each main outcome; consider their relevance to key groups (e.g., healthcare providers, users, and policy makers).	Yes

Limitations	25	Discuss limitations at study and outcome level (e.g., risk of bias), and at review-level (e.g., incomplete retrieval of identified research, reporting bias).	Yes
Conclusions	26	Provide a general interpretation of the results in the context of other evidence, and implications for future research.	Yes
**FUNDING**			N/A
Funding	27	Describe sources of funding for the systematic review and other support (e.g., supply of data); role of funders for the systematic review.	N/A

From: Moher D, Liberati A, Tetzlaff J, Altman DG, The PRISMA Group (2009). Preferred Reporting Items for Systematic Reviews and Meta-Analyses: The PRISMA Statement. PLoS Med 6(7): e1000097. doi:10.1371/journal.pmed1000097

For more information, visit: **www.prisma-statement.org**.
